# Glia: A Neglected Player in Non-invasive Direct Current Brain Stimulation

**DOI:** 10.3389/fncel.2016.00188

**Published:** 2016-08-08

**Authors:** Anne-Kathrin Gellner, Janine Reis, Brita Fritsch

**Affiliations:** Department of Neurology, University Hospital FreiburgFreiburg, Germany

**Keywords:** astrocyte, microglia, tDCS, non-invasive electrical brain stimulation, motor cortex

## Abstract

Non-invasive electrical brain stimulation by application of direct current (DCS) promotes plasticity in neuronal networks *in vitro* and in *in vivo*. This effect has been mainly attributed to the direct modulation of neurons. Glia represents approximately 50% of cells in the brain. Glial cells are electrically active and participate in synaptic plasticity. Despite of that, effects of DCS on glial structures and on interaction with neurons are only sparsely investigated. In this perspectives article we review the current literature, present own dose response data and provide a framework for future research from two points of view: first, the direct effects of DCS on glia and second, the contribution of glia to DCS related neuronal plasticity.

## Introduction

Non-invasive electrical brain stimulation by means of transcranial direct current stimulation (tDCS) is well known for a polarity-specific modulation of cortical excitability and neuroplasticity (Nitsche and Paulus, 2000; Fritsch et al., 2010; Ranieri et al., 2012). Per definition, anodal tDCS refers to placement of the anode above the cortical region targeted for modulation. With stimulation durations exceeding ∼5 min anodal tDCS exerts a transient excitatory effect onto a particular brain region outlasting the stimulation duration for minutes to several hours. Enhanced excitability after anodal tDCS has been observed from cellular to systems level, e.g., as changes in neuronal firing rates (Bindman et al., 1964) or increased motor evoked potentials (Nitsche and Paulus, 2000, 2001). However, since tDCS only minimally changes the neuronal membrane potential (∼1 mV, Purpura and McMurtry, 1965) the modulatory effect on excitability is not yet fully explained. tDCS-increased excitability in combination with an additional synaptic activation may lead to synapse specificity as a source for changes in synaptic strength, which may then produce long-term plastic changes associated with learning and memory (Reis et al., 2009; Fritsch et al., 2010; Ranieri et al., 2012; Rohan et al., 2015).

Processing of information in the brain is traditionally viewed to rely on neuronal action. This view has been challenged by a wealth of evidence supporting glia’s role in neural transmission and neuroplasticity. Glia comprise about 50% of the human brain cells (Azevedo et al., 2009). While they cannot generate action potentials, their cellular properties potentially allow for sensitivity to voltage changes, which may include external electrical stimulation. A role of astrocytes in synaptic plasticity is well established and the underlying signaling cascades have been greatly substantiated (Halassa and Haydon, 2010; Pannasch et al., 2011). In contrast, the participation of microglia in these processes emerged just recently, outdating the concept of the tripartite synapse (i.e., the interaction of astrocytes with the pre- and post-synapse) and leading to the “quadpartite synapse” that includes the participation of microglia (Schafer et al., 2013).

Direct modulation of glial function by DCS and secondary effects on neuronal plasticity are conceivable. Here, we summarize reported DCS effects on glia. We present dose-response data on astroytic and microglial reactivity after anodal tDCS in relation to the occurrence of neurodegeneration. Finally, putative pathways involved in neuroglial plasticity as potential target for DCS modulation are reviewed, providing a framework for future research.

## General Responses of Glia to Electrical Fields

It is long-established that irrespective of their origin or electrical properties cells respond to electrical fields (Ingvar, 1920). Research in this area was largely driven by developmental interests. Remarkably strong endogenous electrical fields in the order of 1–1000 mV/mm occur across the whole embryo and across the neural plate and tube (Metcalf et al., 1994; Nuccitelli, 2003), that are functionally relevant for physiological neurodevelopment. External disturbances of these fields lead to severe developmental abnormalities (Metcalf and Borgens, 1994). Cellular alignment in the electrical field, migration and sprouting of cell protrusions including its directionality are crucial for the integration of cells into the whole system.

Neurons are the most extensively investigated brain cells due to their electrical properties. The common findings of several studies applying external electrical fields, mainly to cultured neurons, are enhanced neurite outgrowth with retraction of neurites facing the anode and increased sprouting toward the cathode (Hinkle et al., 1981; Bedlack et al., 1992; Erskine et al., 1995; Pelletier et al., 2015). Comparability to human tDCS application is lacking, given much longer stimulation durations (hours to days versus 10–40 min) and higher field strengths (7–500 mV/mm versus <1 mV/mm; Miranda et al., 2006; Datta et al., 2009). However, Wood and Willits (2006) reported overall increased neurite sprouting in cultured embryonic dorsal root ganglia after 10 min DCS with relatively low intensity (25 mV/mm) outlasting the stimulation for at least 48 h, but with loss of directionality, most likely due to absence of guidance effects of the electrical field after cessation of stimulation.

Less is known about the direct DCS effects on glia. While protrusion elongation occurs already at low field strength in astrocyte (25 mV/mm) and microglia cell lines (4 mV/mm and 25 mV/mm), alignment occurs at higher intensities (Borgens et al., 1994; Alexander et al., 2006; Bani, 2014; Pelletier et al., 2015). It is interesting to note that astrocytes align perpendicular, but microglia aligns parallel to the electrical field. Migrational behavior was either not observed or not investigated in these studies. Lipopolysaccharide activated microglia shows higher vulnerability to DCS in terms of decreased survival, dose dependent reduction of protrusions and missing alignment at any intensity (Pelletier et al., 2015).

While microglia responds to the electric field by morphological changes, generally indicating activation, markers of neuroinflammation/-degeneration like TNF-alpha, iNOS and interleukins do not increase at any intensity between 4 and 400 mV/mm. This is in accordance with a lack of response to DCS in a phagocytosis assay. As an exception, the pro-inflammatory COX-2 increased in ramified microglia at 100 mV/mm only (Bani, 2014; Pelletier et al., 2015). Glial mediators of neuroprotection like neurotrophic factors have not been investigated in this context. Cultured cortical mouse astrocytes show increased glucose metabolism with low intensity (0.3 mV/mm effective field) and brief 10–30 min DCS (Huang et al., 1997).

Taken together, cultured glia presents cell type and activation state specific morphological responses to DCS with parameters not matching the human conditions. Morphological changes in microglia even at higher intensities are not associated with an activated M1 phenotype, while neuroprotective properties need further investigation. There is evidence for a metabolic response of astrocytes to weak and brief stimulation.

In control experiments of recent *in vivo* studies the response of glia to DCS was investigated to assess inflammatory side-effects (Wachter et al., 2011; Rueger et al., 2012; Peruzzotti-Jametti et al., 2013). Compared to a cell culture where the cells are equally exposed to the anode and cathode (except for Huang et al., 1997), polarity specific stimulation effects occur *in vivo* depending on the polarity of the electrode placed above the region of interest.

**FIGURE 1:**
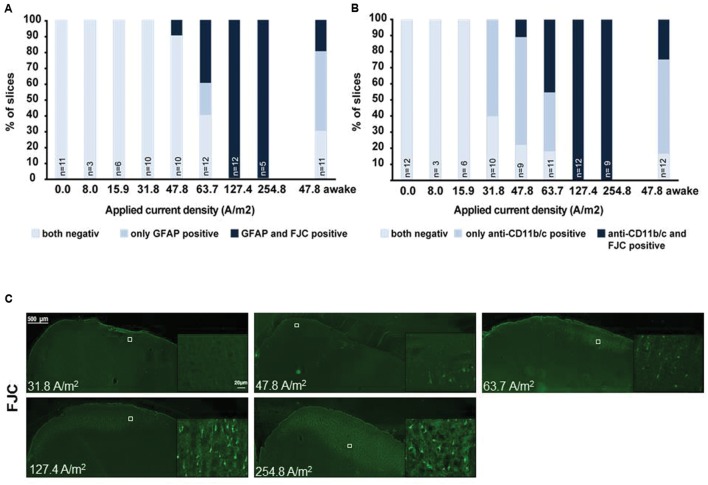
**Relationship of astrocytic, microglial reaction to neuronal damage in brain slices obtained after different doses of anodal tDCS applied to the primary motor cortex.** **(A)** Relation between astrocytic reactivity assessed by GFAP staining (rating: reactive astrocytes = GFAP positive) and neurodegeneration revealed by Fluoro-Jade^®^ C (FJC) positivity. Slices were rated by a blinded investigator either for both GFAP and FJC negative, as GFAP positive only or as both GFAP and FJC positive, GFAP positivity (reactive astrocytes) did not occur in FJC negative slices. Therefore, astrocytic activation occurred only at intensities at which neurodegeneration was observable. **(B)** Relation between morphologically activated microglia assessed by anti-CD11b/c staining (rating: activated microglia = anti-CD11b/c positive) and neurodegeneration revealed by FJC positivity. Slices were rated by a blinded investigator either as anti-CD11b/c and FJC staining negative, as anti-CD11b/c positive only or as both anti-CD11b/c and FJC positive. Note that microglial activation clearly preceded occurrence of neurodegeneration. **(C)** Examples of brain slices exposed to different intensities of anodal tDCS applied to the primary motor cortex. Note no signs 31.8 A/m^2^ of neurodegeneration, while few degenerating neurons are present at 47.8 A/m^2^ and neuronal damage further increases with increasing dose.

*Post hoc* histological assessment of the rat cerebral cortex after i*n vivo* high intensity anodal and cathodal transcranial DCS revealed activation of microglia (Rueger et al., 2012). The stimulation intensity (142.9A/m2 over 15 min = 12.86C/cm^2^) is >150 times higher than in the human application and >2 times above the lowest stimulation intensity inducing neurodegeneration in our own and others safety studies of rat motor cortical tDCS [5.73C/cm^2^ (see below); 5.24C/cm^2^ and below at 2.57C/cm^2^, respectively, (Liebetanz et al., 2009; Wachter et al., 2011)]. Hence, direct effects of tDCS on microglia cannot be separated from secondary activation due to neuronal damage. In rodent stroke, a pathological condition associated with microglial activation, inconsistent results arose: While DCS applied at an intensity causing microglia activation even in the absence of stroke (142.9A/m^2^) pronounced microglia activation and a shift toward a neuroinflammatory phenotype in rats (Braun et al., 2016), lower intensity DCS (55A/m^2^) suppressed microglia activation in mice (Peruzzotti-Jametti et al., 2013). To elucidate dose-dependent direct DCS effects on glia by *in vivo* tDCS we exposed adult male naïve Sprague Dawley rats to 20 min of several doses of anodal tDCS under light iosoflurane anesthesia (see Supplementary Methods). Reactivity of astrocytes and microglia as well as neurodegeneration was assessed by morphological analysis. Neither glia activation nor neurodegeneration was observed at intensities of 15.9A/m^2^ or below (**Figure 1**).

**FIGURE 2:**
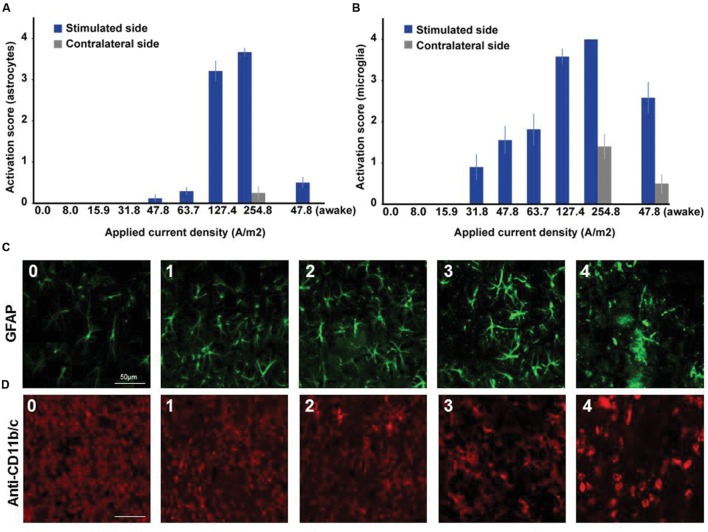
**Dose dependent reactivity of astrocytes and activation of microglia in brain slices obtained after different doses of anodal tDCS applied to the primary motor cortex.** **(A)** Reactivity score (range 0–4) of astrocytes as indicated by histological findings in GFAP staining. With increasing dose, the level of astrocytic activation increased and also affected the unstimulated hemisphere at the highest dose. **(B)** Activation score (range 0–4) of microglia as indicated by histological findings in anti-CD11b/c staining. Starting already at moderate doses the level of microglia activation increased with dose and also affected the unstimulated hemisphere at the highest dose or in awake animals. **(C)** Histological sample images of level of astrocytic reactivity, ranging from 0 (no reactivity) to 4 (severe reactivity). **(D)** Histological sample images of level of microglia activation, ranging from 0 (not activated) to 4 (severely activated).

In microglia, but not in astrocytes, morphological changes occurred at intensities below the threshold for neurodegeneration (31.8 A/m^2^). Severity rating of morphological changes (grade 0–4, **Figure 2**) revealed a dose dependent effect (**Figures 1** and **2**). As expected, glia activation accompanied neurodegeneration in animals subjected to the two highest intensities (127.4 and 254.8A/m^2^). In awake animals tDCS at 47.8A/m^2^ led to slightly higher rates of neurodegeneration and glia activation compared to the anesthetized rat, likely due to lack of excitation suppression by the anesthetic and thus slightly increased excitotoxicity. Astrocyte reactivity only occurred in conjunction with neurodegeneration, while additional dose dependent morphological changes of microglia were independent of neurodegeneration.

In summary, lasting (≥24 h) activation independent of neurodegeneration only occurs in microglia but is likely not relevant at the low intensities applied in humans. High intensity tDCS may have dose dependent differential effects on post-stroke activated microglia. Transient effects in the range of minutes to hours or even during stimulation cannot be excluded from the data available.

## DCS Supported Neuroplasticity – Potential Role of Changes in Glia Physiology

To interact with neurons in basal and plasticity-related activity glia needs to be equipped with sensors and effectors of neurotransmission. For the purpose of this review, we focus on those mechanisms unveiled to date that are potentially sensitive to DCS.

### Astrocytes

The sensitivity of astrocytes to synaptic activity is realized by potassium uptake/buffering through ion channels, by glutamate uptake through transporters and activation of metabotropic glutamate receptors (Dallérac et al., 2013). Inward-rectifying potassium channels (Kir4.1) placed at distal processes close to synapses (Hibino et al., 2004) maintain the strongly hyperpolarized resting potential of astrocytes (∼-80 mV) close to the Nernst equilibrium of potassium (Olsen et al., 2006; Chever et al., 2010). Hence, astrocytes demonstrate high sensitivity to even slight extracellular potassium changes resulting from neuronal activity (Amzica and Massimini, 2002). In mature cortical and hippocampal astrocytes membrane depolarization of 1–2 mV (Orkand et al., 1966; Amzica et al., 2002) accompanies physiological neuronal activity (Djukic et al., 2007; Bernardinelli and Chatton, 2008). *In vivo*, artificial direct depolarization of astrocytes equipped with a light gated unselective cation channel leads to glutamate release and thus to neuronal activity (Sasaki et al., 2012). The relevance of these membrane depolarizations for physiological processes is yet unclear. However, astrocytes express voltage sensitive channels and transporters further supporting functional relevance of membrane potential changes (Barbour et al., 1988; Dallérac et al., 2013), as has been shown for astrocyte metabolism (Orkand et al., 1973; Ruminot et al., 2011) and gap junction coupling (Enkvist and McCarthy, 1994; Roux et al., 2011). Gap junctions, built by connexines, contribute to extensive astrocytic connectivity. Calcium is the downstream mediator connecting the sensing and effector mechanisms in astrocytes. Calcium permeability of gap junctions allows for distribution of calcium through the whole astroglial network (Hirase et al., 2004), and thereby for modulation of wide range neuronal activity (Poskanzer and Yuste, 2011). In accordance, knockout or chemical blockage of connexines seems to be associated with impaired plasticity and learning (Frisch et al., 2005; Hosseinzadeh et al., 2005). Calcium signaling mediated secretion of neuroactive substances (gliotransmitters) like D-serine (a co-agonist at the NMDA-receptor), ATP and/or glutamate promotes *in vitro* long-term potentiation (LTP) of synaptic transmission in hippocampus and neocortex (Henneberger et al., 2010; Navarrete et al., 2012; Pannasch and Rouach, 2013; Pankratov and Lalo, 2015). Corroborating these results, *in vivo* motor learning is impaired in mice with deficiency of intra-astrocytic calcium signaling, which can be partially rescued by supplementation of D-serine (Padmashri et al., 2015). Human cortical astrocytes engrafted to mice enhanced LTP and multimodal learning (Han et al., 2013). These cells are larger with fourfold faster calcium signaling than in rodents (Oberheim et al., 2009) suggesting that the magnitude of astrocytic calcium rise affects LTP expression. Accordingly, *in vivo* tDCS (50A/m^2^) induces a metaplastic potentiation of somatosensory evoked potentials dependent on calcium/inositol triphosphate signaling. This was mediated by noradrenergic transmission, with the source of noradrenaline secretion not investigated but supposedly attributed to neuronal activity (Monai et al., 2016). Secondary to neuronal activity also structural changes, swelling attributed to potassium buffering, may occur (Ballanyi et al., 1987). Astrocytic swelling leads to a reduction of extracellular space, which increases ephaptic excitation (field effects) and the concentration of extracellular neuroactive molecules and ions, e.g., glutamate and potassium, secondarily promoting excitation (Rosen and Andrew, 1990; Syková, 2004).

Taken together, astrocytes are multimodally equipped to detect even slight neuronal activity and integrate this information through several mechanisms. Despite that, dynamics of astrocytic membrane potential changes under the influence of DCS have not been assessed. Direct measurements with and without intact neuronal firing (e.g., accomplished by application of tetrodotoxine) are desirable to distinguish primary from secondary effects through neuronal activity. However, physiologically observable astrocytic membrane potential changes in response to neuronal activity (Orkand et al., 1966; Pannasch and Rouach, 2013), are in the range of those theoretically estimated to occur during DCS (Ruohonen and Karhu, 2012). While such estimations of membrane potential changes are based on properties of single astrocytes, it is an intriguing hypothesis that a whole astrocytic network, coupled by gap junctions, may show even greater membrane potential changes due to a larger polarizable substrate. DCS may exert voltage dependent effects on astroglia via voltage sensitive transporters and channels, alteration of gap junction coupling or metabolism with consecutive changes of neurotransmission and neuroplasticity.

### Microglia

For the purpose of reviewing neuron-microglia interaction in adult neuroplasticity we exclude data from embryonic/postnatal cultures and microglia cell lines due to different roles in development and varying physiological properties compared to the adult brain.

#### Ramified Microglia

Assessing the microglia-neuron interaction in basal and plasticity-related neurotransmission has been a major technical challenge, since microglia in its physiological ramified state is not accessible in cell culture preparations (Schilling and Eder, 2007). Recording from acute brain slices represents the closest *ex vivo* approach (Kettenmann et al., 2011). Microglial properties include high input resistance, low membrane potentials (∼-20 to -55 mV) and lack of relevant voltage-gated membrane currents (Boucsein et al., 2000, 2003; Schilling and Eder, 2007, 2015), which was found to be comparable in human ramified microglia in acute slices from biopsies (Bordey and Spencer, 2003). *In vivo* data on the capability of ramified microglia to modulate basal neuronal or astrocytic activity and transmission are to our knowledge not available. However, the role of ramified microglia in pruning and maturation of synaptic connectivity during neurodevelopment, processes also relevant for adult neuroplasticity, is well established (Kettenmann et al., 2013; Schafer et al., 2013). *In vivo* 2-photon imaging of fluorescent neocortical microglia (Jung et al., 2000) revealed responses to experience dependent changes in neurotransmission with directed process motility toward synapses with contacted once tended to be pruned (Wake et al., 2009; Tremblay et al., 2010; Schafer et al., 2013). Increased process motility has been attributed to excitation and inhibition to reduced motility. Enhanced process motility is assumed to result from glutamate-mediated ATP signaling, the latter secreted by astrocytes (Davalos et al., 2005; Nimmerjahn et al., 2005; Fontainhas et al., 2011). However, opposite effects on motility have also been described (Grinberg et al., 2011). Fractalkine (CX3CL1) signaling from neurons to microglia, which exhibit the fractalkine receptor (CX3CR1), is suggested to be a crucial mediator of adult memory formation. Knock-out of the microglia-specific fractalkine receptor Cx3CR1 results in impaired LTP in the hippocampus and associated learning deficits (Rogers et al., 2011), while LTD is not affected in adult CX3CR1 deficient mice (Paolicelli et al., 2011). A role in hippocampal homeostatic plasticity is suggested by diminishing effects of CX3CR1 depletion on priming of LTP through enriched environment. Interestingly, a weak increase of synaptic strength was seen in these mice without environmental priming (Maggi et al., 2011). Inducible depletion of microglia during adulthood, excluding adaptations during development, results in deficits in multiple learning tasks and disturbed spine dynamics attributed to the lack of microglial BDNF secretion and activation of downstream cascades via TrkB (Parkhurst et al., 2013). Taken together, a change in membrane potential is unlikely to be of physiological relevance in ramified microglia. Taking the rather radial shape of microglia into account, changes in membrane potential below those observed in pyramidal neurons or astrocytes can be assumed (Radman et al., 2009), but still need further investigation. An intermediate role in DCS related plasticity in response to neuronal or astrocytic activity changes is conceivable.

#### Amoeboid Microglia

In contrast to the sparse data in ramified microglia, sensing of neurotransmission, e.g., for ACh, glutamate, GABA, and ATP is well documented for amoeboid microglia, as well as cons ecutive changes in intracellular calcium, membrane potential, cytokine release, and motility (Biber et al., 2007; Kettenmann et al., 2011). Moreover, amoeboid microglia hold a larger degree of voltage sensitive currents (Kettenmann et al., 2011). The neuroinflamatory/cytotoxic M1 phenotype is associated with neurodegenerative diseases such as Alzheimer’s disease, characterized by impaired learning, and memory, which is likely related to the release of pro-inflammatory/cytotoxic factors like TNF-alpha, interleukin-1beta and -6 by M1 microglia (Lynch et al., 2004; Tang and Le, 2015). Of note, interleukin-1beta has been implicated in both promotion and disturbance of hippocampal LTP (Rogers et al., 2011; Nisticò et al., 2014). The relationship between and interaction of microglia activation, inflammation and involvement in neuroplasticity is still a field of controversy and largely under investigation. Interleukin-4 and -10 as well as trophic factors like BDNF and IGF-I secreted by the neuroprotective M2 phenotype have in general been shown to augment LTP and/or behavioral learning (Lynch et al., 2004; Fritsch et al., 2010; Gadani et al., 2012; Burgdorf et al., 2015; Tang and Le, 2015). Taken together, amoeboid microglia might be more susceptible to DCS as they are well equipped with voltage gated ion-channels. With regard to Parkhurst’s data and a critical role of BDNF secretion and TrkB receptor phosphorylation in DCS mediated synaptic plasticity (Fritsch et al., 2010) it is tempting to speculate that M2 microglia might support plasticity relevant for neurorehabilitation and network re-organization in the diseased brain.

## Final Remarks

In non-invasive brain stimulation research glia – a potential polarizable cell type and contributor to adult neuroplasticity – can no longer be neglected. Cell culture studies are technically limited, but can provide supportive information on DCS effects. To date, the investigation of non-combined cultures to assess cell-type specific DCS effects is lacking. Advanced *in vivo* imaging techniques allow for assessment of glia in its physiological environment in the absence and presence of external stimuli and display a highly promising tool to study DCS-related neuroplasticity. Lastly, future research approaches aiming at understanding the contribution of glia to (t) DCS-related plasticity should be closer related to protocols used in humans in terms of stimulation intensities and durations to ease translation into the clinical context.

## Author Contributions

All authors listed, have made substantial, direct and intellectual contribution to the work, and approved it for publication.

## Conflict of Interest Statement

The authors declare that the research was conducted in the absence of any commercial or financial relationships that could be construed as a potential conflict of interest.
