# Supplementary material for: Glia: A Neglected Player in Non-invasive Direct Current Brain Stimulation
Source: Front Cell Neurosci. 2016 Aug 8;10:188. doi: 10.3389/fncel.2016.00188 (PMC4976108; doi:10.3389/fncel.2016.00188)
Supplement: Supplementary file 1 [file Data_Sheet_1.PDF]

# Glia: A Neglected Player in Noninvasive Electrical Brain Stimulation

**Anne-Kathrin Gellner[1], Janine Reis[1], Brita Fritsch[1]***

## Supplementary Methods

### Animals

All animal work was performed according to the Animal Protection Law and the Directive 2010/63/EU of the European Commission. The animal proposal was approved by the "Commission for Animal Experimentation of the Regional Council of Freiburg" and "Commission for Animal Experimentation of the University Medical Center". Adult, 8 weeks old male Sprague-Dawley rats (Charles River, Sulzfeld, Germany) were used and housed under standardized conditions including a 12 hours light/dark cycle, temperature at 21±1°C and food and water ad libitum.

### Surgery and tDCS

Under sufficient isoflurane anesthesiathe animal was placed in a sterotactic frame. To ensure a standardized electrode contact area a plastic screw tube (4 mm inner diameter) was fixed with acrylic cement to the skull above the left primary motor cortex (center: mm from Bregma: A/P = 2.0, M/L = 2.0). For stimulation, the level of anesthesia was reduced (0.5-1%, O2 1l/min) so that a slight toe pinch response was present. A tDCS electrode (Ag/Ag-Cl, contact area of 12.56 $mm^2$; World Precision Instruments Inc., Sarasota, FL, USA and in-house production) was inserted into the saline filled screw tube and a conductive rubber counter electrode (6 $cm^2$) was placed on the animal's chest. Anodal stimulation intensities were sham, 8, 15.9, 31.8, 47.8, 63.7, 127.4 or 254.8 $A/m^2$ applied for 20 minutes using a 9V battery-driven stimulator with ramp-up function (in-house production). Level of anesthesia, vital parameters and current flow were constantly monitored. After tDCS rats were allowed to wake up and recover. To take interfering effects of the anesthesia into account awake rats were also tested at the lowest intensity at which sparse neurodegeneration was detected under anesthesia (47.8 $A/m^2$). These rats were familiarized with the tDCS stimulation setup, for which the counter electrode was placed on the animal's chest and fixed by a vest.

### Histological Analysis

Twenty-four hours after tDCS animals were sacrificed under deep anesthesia (ketamine/xylazine) by cardiac perfusion with ice cold phosphate buffered saline pH 7.4 (PBS) followed by 4% paraformaldehyde. Brains were immediately removed, postfixed with 4% paraformaldehyde at 4°C for 24 hrs and then transferred to 0.1 M PB containing 30% sucrose as cryoprotection for another 2 days. Afterwards the samples were cut into serial coronar sections (30 µm) using a frozen sliding microtome.

To visualize degenerative neurons, brain sections were mounted on gelatin-coated slides, air-dried, and subjected to FJC staining strictly following the protocol of Schmued et al. (Schmued et al., 2005): Slides were immersed subsequently in solutions of 1% NaOH in 80% ethanol (5 min), 70% ethanol (2 min) and distilled water (2 min) followed by incubation in 0.06% potassium permanganate (10 min), destilled water (2 min) and FJC staining solution (10 min). The proper dilution was accomplished by first making a 0.01% stock solution of FJC dye (Chemicon, Temecula, CA, USA) in distilled water and then adding 1 ml of the stock solution to 99 ml of 0.1% acetic acid. Slides were washed three times each for 1 min, air-dried on a slide warmer at 50°C for 30 min and after clearing in xylene coverslipped with DPX (Thermo Fisher Scientific, Waltham, MA, USA).

Immunolabeling was used to characterize changes in astrocytes and microglia morphology using antibodies against glial fibrillary acidic protein (rabbit anti-GFAP, Sigma Aldrich, St. Luis, MO) and CD11b/c (mouse anti-CD11b/c, Biolegend, San Diego, CA) respectively. A series of free floating sections of each animal was rinsed in 0.5% Tween in PBS 3x10 min, transferred to a blocking solution (3% donkey serum, 0.1% Tween in PBS) for 30 min at room temperature an then incubated at 4°C for 24 h with either anti-GFAP 1:500 or anti-CD11b/c 1:500 diluted in 3% donkey serum and 5% bovine serum albumin in 0.1% Tween in PBS. Sections were washed again as described above and incubated with the appropriate secondary IgG antibody (Alexa 488 anti-rabbit or Alexa 555 anti-mouse, Invitrogen, Carlsbad, CA, USA) for 60 min at room temperature. Afterwards 3 washing steps followed. Sections were then mounted onto slides, dried overnight at room temperature and then coverslipped using ProLong Gold Antifade Mountant (Thermo Fisher Scientific, Waltham, MA, USA).

## Evaluation and Scoring System

For each staining technique, three brain sections (+3, 0 and -1.56 mm AP from Bregma) per animal were selected. For FJC staining we used an epifluorescence microscope (Axioplan 2, Zeiss, Oberkochen, Germany) and its fluorescein/FITC filter system and acquired images of both the (sham-)stimulated and control hemisphere with an 10x objective. Immunostainings were imaged using a confocal laser scanning microscope and its appropriate filter settings (FV10i, Olympus, Tokyo, Japan). Z-stacks within layer II/III of the motor cortex of both hemispheres were captured at 60x magnification.

Neurodegeneration was rated binary for FCJ staining of neurons being present or absent. Glia was rated by a modified grading method by Blackbeard et al.: glial activation led to a gradual thickening and shortening of processes, hypertrophy of cell bodies and uneven distribution. In the most severe stage cell debris was predominant.

## References

Schmued L. C., Stowers C. C., Scallet A. C., Xu L. Fluoro-Jade C results in ultra high resolution and contrast labeling of degenerating neurons. Brain Res. 2005;1035:24–31

J. Blackbeard, K.P. O’Dea, V.C.J. Wallace, A. Segerdahl, T. Pheby, M. Takata, M.J. Field, A.S.C. Rice, Quantification of the rat spinal microglial response to peripheral nerve injury as revealed by immunohistochemical image analysis and flow cytometry, Journal of Neuroscience Methods, Volume 164, Issue 2, 30 August 2007
